# Development of a resource use measure to capture costs related to unpaid care for people living with non-memory led dementia: a modified Delphi study

**DOI:** 10.1136/bmjopen-2025-110399

**Published:** 2026-02-12

**Authors:** Katherine Cullen, Emilie V Brotherhood, Oliver Hayes, Valerie Mansfield, Aida Suarez-Gonzalez, Nikki Zimmermann, Joshua Stott, Deborah Fitzsimmons

**Affiliations:** 1 Swansea Centre for Health Economics, Faculty of Medicine, Health and Life Sciences, Swansea University, Swansea, UK; 2 Dementia Research Centre, Queen Square Institute of Neurology, University College London, London, UK; 3 ADAPTlab, Research Department of Clinical Educational and Health Psychology, University College London, London, UK

**Keywords:** Dementia, Caregivers, Surveys and Questionnaires, Health Care Costs, HEALTH ECONOMICS

## Abstract

**Abstract:**

**Objectives:**

To determine the personal, National Health Service and wider societal resource use in relation to caring responsibilities for carers of people living with non-memory led dementias (NMLDs); and to design a resource use measure (RUM) that can be delivered in the Better Living with Non-memory-led Dementia (BELIDE) randomised controlled trial, part of the Rare Dementia (RD) - TALK research programme.

**Design:**

The first stage was to identify and review any existing RUMs that could be used or adapted to the trial population and setting. If no measures were identified, the second stage was initial informal discussions with healthcare professionals (HCPs) and the programme patient and public involvement representatives to inform the perspective, settings of care and main resource items to develop a new RUM. In the third stage, a first draft of the RUM was tested for content and face validity in a modified Delphi study comprising HCPs and carers. The measure was revised and, in the final stage, piloted in the first 3 months of the BELIDE trial to assess acceptability and feasibility of collecting the economic outcomes and the completeness of data collection. The key drivers of resource use and costs were assessed, and appropriate face validity checks were applied to ensure accurate description of the treatment pathways.

**Participants and setting:**

Carers and family of people living with NMLD recruited from Rare Dementia Support members in the UK, and a broad range of HCPs with experience of working with people who have NMLD to capture the different dimensions of experience, grade and skill mix.

**Results:**

In total, 20 people participated in the modified Delphi study, 11 HCPs and 9 carers. Rare Dementia Support groups and 1:1 calls were highly rated, as were general practitioner appointments. The greatest consensus was in the productivity and carer tasks; all caring tasks were highly rated. Healthcare practitioners rated healthcare items as higher importance than carers themselves.

**Conclusions:**

Unpaid carers and HCPs are the experts in the resource impact of caring for someone with NMLD and have been underserved in research to date. This research, as part of preparatory stages of the BELIDE trial, has enabled the timely development of a comprehensive and valid RUM for unpaid carers of people with NMLD.

**PROSPERO registration number:**

CRD42022356943.

**Trial registration number:**

NCT06241287.

**STRENGTHS AND LIMITATIONS OF THIS STUDY**

- Co-developing a resource use measure (RUM) with carers of people with non-memory led dementia to identify resource items relevant to their distinct needs.
- The RUM was tested in an embedded pilot study to determine if the measure was written in a clear and understandable way to allow accurate responses.
- A pragmatic approach was used with convenience sampling to use existing networks to recruit study participants, and this may not capture a fully representative population.
- A challenge for this population was distinguishing between services used by themselves in a caregiving capacity and those accessed on behalf of the person with dementia, and those accessed in a non-caregiving capacity.
- Developing an RUM with carers and healthcare professionals informs the feasibility and relevance of the measure.

## Introduction

 In 2023, an estimated 18.4 billion hours of unpaid care was provided for people with dementia in the USA valued at US$346.6 billion.^1^ Those affected by non-memory led dementias (NMLDs), such as posterior cortical atrophy, primary progressive aphasia or behavioural variant frontotemporal dementia, face additional challenges to those associated with ‘typical dementias’ including unusual symptoms, such as language difficulties and personality changes, which can affect behaviour and social functioning, and visual decline.^2^,^4^ With a younger average age at onset, people living with these dementias are more likely to have school-aged children or dependent parents, with family members often becoming the primary income earner alongside caring for the person living with dementia. Half of primary carers for people with dementia are employed, but many need to leave employment or cut their working hours, which can reduce their standard of living.^5^ Carers UK estimates that one in seven people in the workplace are balancing work with caring responsibilities.^6^ Where there is a significant impact on the health and well-being of those involved in a patient’s care by not including this in evaluations, we will not reflect the true value of treatments and interventions.^7^ As part of the Rare Dementia (RD) - TALK research programme (National Institute for Health and Care Research (NIHR) 203680), a web-based blended care self-management intervention has been developed for carers of people with NMLDs; the effectiveness and cost-effectiveness will be assessed in the Better Living with Non-memory-led Dementia (BELIDE) randomised controlled trial (RCT) (NCT06241287).^8^

The UK Medical Research Council has published guidance on evaluation of complex interventions which highlights the essential role that people with experience of a condition have in identifying which outcomes to measure.^9^ Involving people with lived experience of a condition in choosing the perspective for an evaluation and the economic outcomes, such as the resource use items to collect, ensures we are capturing the most relevant outcome data. Economic evaluation requires the collection of participant level resource use (such as doctor appointments, hospital attendances and out-of-pocket expenses) associated with the intervention compared with usual care. There is currently no standard methodology in how resource use data should be collected for health economic evaluation, which leads to inconsistency across studies. While routine data, for example, hospital episode statistics in the UK,^10^ may provide information on health service use and possibly social care use, it will not present the complete picture of resource use for carers who may incur significant personal costs related to caring responsibilities. Resource use data can be collected directly from the study participants using a questionnaire, diary or interviews. Frequently, the client-services receipt inventory (CSRI) is adapted by a research team, but without discussion with people with experience of a condition or reporting the process used to make changes,^11 12^ which will limit content validity. A systematic review of methods for collection of resource use data within clinical trials found only a minority of studies employed a systematic approach for resource identification at the planning stage, even though this is a critical stage to identify the main resource drivers and associated costs.^13^ Although the inclusion of outcomes relevant for unpaid carers is considered key for comprehensive economic evaluation of interventions for dementia, there is currently no consensus on how to include outcomes for unpaid carers.^14^

To ensure we are collecting the key drivers of resource use and costs for this population, an initial stage of this research has been to develop a suitable measure and to consider approaches to identify, measure and value resource use in monetary terms to inform health economic analysis. However, this work often is required within time constraints within setting up a trial, thus we had to balance the need for a robust process to produce a suitable resource use measure (RUM) with the demand to deliver without delay to the RD-TALK programme. We thus built in a preparatory work package to the RD-TALK programme grant to identify, develop and pilot a suitable RUM that could be used within the BELIDE trial.

### Objectives

Our objectives were to (1) identify any candidate RUMs; (2) gather information from the perspective of carers for people with NMLD and relevant stakeholders on the resources and associated costs for carers, the National Health Service (NHS) and wider society; and (3) to design an RUM that could be delivered in the trial.

## Methods

### Study design

The RUM should be as short as possible to reduce participant burden focusing on capturing the key, relevant items of interest but sufficiently comprehensive to collect the key drivers of resource use and costs for this population.^15 16^ It is also important to be sensitive to the needs of the participants, especially when related to sensitive topics such as caregiving.^15^

The first stage was to determine if a suitable measure was already available that could be used or adapted to the trial population and setting. Second was the planning stage, involving discussion with healthcare professionals (HCPs) and patient and public involvement (PPI) leads from the RD-talk programme to determine the perspective, population, cost-drivers and settings of care, and main resource items. In the third stage, the first draft of the RUM was tested for content and face validity by HCPs and carers in a modified Delphi study. And lastly, the RUM was revised and piloted to assess acceptability and feasibility of collecting the resource items and the completeness of data collection. The key drivers of resource use and costs were described, and appropriate face validity checks were applied, with input from the PPI and RD-TALK research team, to ensure accurate description of the treatment pathways.

#### Stage one: literature search

The Database of Instruments for Resource Use Measurement^17^ and the NIHR library^18^ were searched to identify any relevant methods of collecting resource use in similar UK trials, or in a population with rare dementia. A rapid literature review was conducted to identify evaluations of psychological interventions for people living with dementia and their carers as part of the overall RD-TALK programme grant to determine the most appropriate method for evaluation (PROSPERO CRD42022356943, online supplemental material). Electronic searches of seven databases: MEDLINE, PubMed, the Cochrane Library, PsycINFO, CINAHL, NHS EED (up to 31 March 2015) and INAHTA were conducted in November 2022 using key words for adults aged 18 years and older with dementia and carers of people living with dementia. Only titles and abstracts, with full text available in English, were included. All records were screened by one reviewer (KC), with 10% of records screened by a second reviewer (DF) to check for validity. Measures for collecting resource use were identified from the included records.

#### Stage two: initial discussions

**Figure 1:**
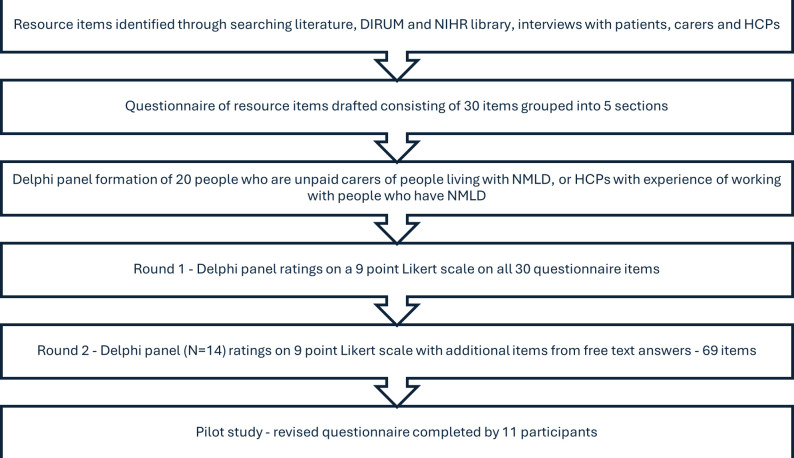
Flow chart of the resource use measure development process. DIRUM, Database of Instruments for Resource Use Measurement; HCP, healthcare professional; NIHR, National Institute for Health and Care Research; NMLD, non-memory led dementia.

A pragmatic approach was applied to the initial stages taking advantage of the established research team, including HCPs and PPI leads from the RD-TALK programme team, to review published measures identified from the literature review to determine if they were suitable for use in the trial (figure 1). On agreement that RUMs available were not suitable for this population without adaptation, a list of key resource use items was developed using inputs from the identified published evaluations and discussion with key stakeholders (identified through stakeholder analysis to ensure high interest, high influence stakeholders were involved from inception, ie, the RD-talk researchers, PPI and HCPs working with people with NMLD).^19^ A suitable perspective for the evaluation, based on the BELIDE trial aims, was discussed to determine the resource items to consider, for instance, health and social care resource use and costs only, or a wider perspective including personal costs and impact on productivity.

#### Stage three: modified Delphi study

To ensure the selected items represented a consensus view, we undertook a modified Delphi study.^20^ The participant population consisted of two groups; carers of people with NMLD and a broad range of HCPs with experience working with people who have NMLD to capture the different dimensions of experience, grade and skill mix (eg, nurses, psychologists). Including HCPs as well as carers ensured cognitive diversity in the panel, bringing different knowledge that would complement each other’s expertise.^21^ All participants were aged 18 or older with a sufficient command of English.

Carers and family were recruited from Rare Dementia Support (RDS) members, identified as willing and appropriate to participate in this research by the direct support team lead at RDS and PPI co-lead on the RD-TALK programme grant. The initial approach was by email from the RD-TALK programme manager and research assistants at University College London. Snowball sampling was used where possible, for example, participants could provide the study information to other family members not currently involved in the study. HCPs were recruited from current networks and contacts known to the chief investigator and RD-TALK researchers, the RDS - which provides 16 support groups in London and 30 regionally, as well as other rare dementia, dementia and Alzheimer’s networks who had opted to hear about research opportunities as part of their membership of the RDS. A formal sample size calculation was not required as there is no consensus on the size of the panel for the Delphi process.^22^ The number and characteristics of participants should be carefully considered.^22^ Recent assessment of replicability of results in multistakeholder Delphi surveys found that sample sizes of 60–80 resulted in high replicability, 20–40 participants resulted in moderate replicability.^22^ A greater number of participants enhances the reliability of findings;^23^ however, participant recruitment and retention in Delphi surveys is challenging, especially in rare diseases,^22 24 25^ and small sample sizes can still provide valid and reliable findings,^22^ allowing development of the RUM within the timescales of the trial.

Potential participants were provided with an invitation email (one for HCPs and another for carers and family members), including a link to an online platform where the participant information sheet, consent form and round 1 survey was hosted. A reminder to complete the questionnaire was emailed to each participant if it was not completed after 1 week with a further reminder a week later if needed.

Participants were asked to rate each item on a 9-point Likert Scale according to the level of importance of the item to carers of people diagnosed with NMLD, 1 indicated ‘not important’ and 9 indicated ‘very important’.^26 27^ A 9-point scale allows the inclusion of a ‘neither disagree nor agree’ to identify items where participants have neutral feelings towards them.^17 26 27^ For each item, there was the opportunity to provide comment and to include additional items that the participant considered very important.^28^

### Data analysis

Each item from round 1 was summarised using descriptive statistics (mean, SD, median, range, IQR, percentage agreement). The analysis of the free text responses (ie, potential new items) was undertaken separately at each round. An analysis plan was developed prior to analysis in Stata (StataCorp LLC, V.18) (online supplemental material).

The overall results of the first-round analysis were presented in a table including median score, IQR, lowest and highest ratings, the participant’s response and a summary of all comments received.^29^ Participants were then asked to re-rate all items in the second round.

To avoid overburdening participants, and given the resources available, no more than three rounds were planned if consensus was not reached.^29^ This number of rounds has been consistently used within the literature to yield robust results.^27 28^ Consensus was determined as an IQR of ≤2 units on the Likert scale.^30^ To ensure the most important resource use items were included in the final RUM, the criteria for inclusion were ≥70% participants scoring 7–9 and ≤15% participants scoring 1–3. The criteria for removing items were ≥70% participants scoring 1–3 and ≤15% participants scoring 7–9.^31^0 To ensure that non-response to an item did not bias the analysis, non-responses were excluded from the percentage agreement. Items that did not reach the criteria for inclusion or exclusion were discussed with the research team.

We considered the level of agreement across participants and across rounds and documented the degree of stability in scores. The intraclass correlation coefficient (ICC) test (two-way random effects model with ICC average measurement and absolute agreement between raters) was used to assess inter-rater reliability between rounds for all items to assess agreement among raters with multiple scores for a rater.^32^ Agreement of 0.75 or above was taken as an indication of good reliability, 0.5–0.75 as moderate and 0.5 as poor.^32^

Wilcoxon signed-rank tests were undertaken for the difference in responses between rounds to assess the stability of the raters’ responses.^33^ A p value >0.05 indicated no evidence that the results were different between the rounds.

The results of decision rules applied at each round were presented along with the items that met the inclusion criteria for the final round of analysis. Each item was further considered in terms of relevance to the BELIDE trial populations.

#### Final stage: pilot study

Within the BELIDE randomised waiting list control trial, a pilot of the RUM was embedded to assess the content validity and completion rates in the initial 3 months of recruitment. The multicentre trial includes adults, aged ≥18 years who self-identify as an unpaid carer of someone with primary progressive aphasia, posterior cortical atrophy or behavioural variant frontotemporal dementia who is not living in a full-time care facility. The data collected at baseline for the first 10–15 participants was checked for face validity. Items with missing data were reported, with the proportion of missing data and patterns examined to assess content validity.

### Public and patient involvement

The PPI leads for the RD-TALK programme were key in determining the suitability of the previously published RUMs for this population, identifying key resource use items and selecting the most appropriate perspective.

## Results

### Literature search

The review of economic evaluations of psychological therapies for dementia identified one RUM developed for people with dementia.^34^ The Resource Utilisation in Dementia (RUD) instrument^35^ was designed to collect data on formal and informal care resource use in different care settings, across different countries. The RUD was reviewed by the RD-TALK PPI representatives who commented on the relevance of several items for carers of people living with NMLD, including the categories used for caring tasks (personal and physical care, practical help and managing financial matters, supervising), as the RUD was designed for people with more typical forms of dementia they would more likely be older aged at onset.

Five NIHR reports related to dementia reported health economic evaluations.^36^,^40^ However, four used a modified version of a psychiatric CSRI,^36^,^3941^ one reported using a Health and Social Care Resource Use Questionnaire; however, it was not included in the report or published.^40^ A service use questionnaire was identified from the Database of Instruments for Resource Use Measurement database. Again, this was a modified CSRI.^41 42^

It was decided to develop a new RUM for this trial. Initial resource use items were discussed with the RD-TALK research team and elicited from economic evaluations in dementia as part of the wider review. Items suggested by the research team included healthcare appointments, psychological therapies, support groups and employment. In an investigation of a service to support carers of people with dementia, the 10 tasks from the 2009 Survey of Carers in Households were suggested for the carer tasks which were included as items for the modified Delphi survey with additional carer task items suggested by the research team of help provided in the home, in a residential setting and the carers assessment, and managing challenging behaviour^43 44^ (box 1).

Box 1 **Tasks listed from the 2009 survey of carers in households^43 44^**

- Personal care, for example, dressing, bathing, washing, shaving, cutting nails, feeding, using the toilet.
- Physical help, for example, with walking, getting up and down stairs, getting into and out of bed.
- Helping with dealing with care services and benefits, for example, making appointments and phone calls, filling in forms.
- Helping with other paperwork or financial matters, for example, writing letters, filling in forms, dealing with bills.
- Other practical help, for example, preparing meals, doing their shopping, laundry, housework, household repairs.
- Keeping them company, for example, visiting, sitting with, reading to, talking to.
- Taking them out, for example, for a walk or drive, taking to see friends or relatives, social activities, accompanying to appointments.
- Giving medicines, for example, making sure they take pills, giving injections.
- Keeping an eye on them to see they are all right.
- Other help.

### Modified Delphi study

In total, 20 people expressed an interest and participated in the modified Delphi study, 11 HCPs and 9 carers. Of the 30 items listed in the first version of the RUM, only 9 reached all criteria for agreement to keep, with no items rated to discard (table 1). Several new items were suggested, ranging from alternative therapies such as yoga and aromatherapy, as well as various family and carer support services. All new items were included in round 2 (table 2). In round 2, seven HCPs and seven carers remained. 11 more of the original items and 4 new items reached all criteria for agreement to keep and were considered very important, with no items reaching the criteria for removal (tables1  2).

**Table 1:** Median, IQR, percentage agreement, ICC and Wilcoxon signed-rank p value for resource use items in round 1 and 2 of the modified Delphi study

	Round 1		Round 2			
	Median (IQR)	Percentage agreement	Median (IQR)	Percentage agreement	ICC	Wilcoxon signed-rank p value
*GP face-to-face*	7.5 (5.5–9)	7–9, 70.0 1–3, 0	8 (6–8)	7–9, 92.0 1–3, 0	0.94	0.15
GP online/telephone	6 (5–7)	7–9, 30.0 1–3, 20.0	6 (4–8)	7–9, 38.0 1–3, 23.0	0.56	0.48
Practice nurse face-to-face	7 (5–8)	7–9, 60.0 1–3, 10.0	7 (5–8)	7–9, 62.0 1–3, 15.0	0.95	0.20
Practice nurse online/telephone	5 (3.5–5.5)	7–9, 10.0 1–3, 25.0	5 (5–5.5)	7–9, 17.0 1–3, 17.0	0.55	0.12
Community mental health face-to-face	9 (5.5–9)	7–9, 70.0 1–3, 0	8 (5–9)	7–9, 69.0 1–3, 0	0.99	1
Community mental health online/telephone	7 (5–9)	7–9, 55.0 1–3, 0	6 (5–7)	7–9, 38.0 1–3, 1.0	0.42	0.72
Psychological intervention online/telephone (n=19)	5 (3–7)	7–9, 31.6 1–3, 26.3	5 (4–6.5)	7–9, 25.0 1–3, 17.0	0.79	0.62
*Psychological intervention face-to-face ≤6 sessions (n=19)*	8 (6–9)	7–9, 68.4 1–3, 5.3	7.5 (6–8)	7–9, 75.0 1–3, 1	0.82	0.71
Psychological intervention face-to-face 7+ sessions (n=19)	7 (5–9)	7–9, 57.9 1–3, 5.3	7 (6.5–7.5)	7–9, 75.0 1–3, 0	0.90	0.05
Pharmacy	6.5 (3.5–9)	7–9, 50.0 1–3, 25.0	5 (5–7)	7–9, 31.0 1–3, 23.0	0.90	0.54
*Rare Dementia Services group*	8 (5.5–9)	7–9, 65.0 1–3, 0.0	8 (7–9)	7–9, 83.0 1–3, 0	0.79	0.33
**Rare Dementia Services 1:1 call**	9 (8–9)	7–9, 80.0 1–3, 0	9 (8–9)	7–9, 92.0 1–3, 0	0.88	0.16
Private psychological therapy (n=7)	5 (5–8)	7–9, 28.6 1–3, 0	6 (5–9)	7–9, 40.0 1–3, 0		0.3
**Time off work to attend appointments with a person with NMLD, paid and unpaid (n=18)**	9 (9–9)	7–9, 88.9 1–2, 11.1	9 (9–9)	7–9, 82.0 1–3, 9.0	0.96	0.32
**Time off work/ sick leave, paid and unpaid due to caring for a person with NMLD (n=18)**	9 (7–9)	7–9, 77.8 1–3, 11.1	9 (8–9)	7–9, 82.0 1–3, 9.0	0.89	0.05
*Benefits: carer in receipt of allowance (n=19)*	9 (5–9)	7–9, 68.4 1–3, 5.3	9 (7–9)	7–9, 75 1–3, 0	0.80	0.05
**Early retirement due to caring for someone with NMLD (n=18)**	9 (7–9)	7–9, 83.3 1–3, 11.1	9 (5–9)	7–9, 73 1–3, 18	0.93	0.92
**Personal care**	9 (8.5–9)	7–9, 85.0 1–3, 10.0	9 (8–9)	7–9, 85.0 1–3, 8.0	0.92	0.95
*Physical help*	9 (5–9)	7–9, 60.0 1–3, 20.0	9 (7–9)	7–9, 77.0 1–3, 15.0	0.93	0.39
**Helping with dealing with care services and benefits**	9 (8.5–9)	7–9, 80.0 1–3, 0	9 (9–9)	7–9, 92 1–3, 0	0.82	0.28
*Helping with other paperwork or financial matters*	9 (6.5–9)	7–9, 75.0 1–3, 5.0	9 (8–9)	7–9, 92.0 1–3, 8.0	0.83	0.3
*Other practical help*	9 (6.5–9)	7–9, 75.0 1–3, 5.0	9 (8–9)	7–9, 85.0 1–3, 8.0	0.99	0.32
**Keeping him/her company**	9 (8–9)	7–9, 85.0 1–3, 10.0	9 (7–9)	7–9, 77.0 1–3, 15.0	0.96	0.93
**Taking him/her out**	9 (7.5–9)	7–9, 90.0 1–3, 10.0	9 (8–9)	7–9, 85.0 1–3, 15.0	0.99	0.63
**Giving medicines**	9 (8–9)	7–9, 80.0 1–3, 5.0	9 (9–9)	7–9, 92.0 1–3, 8.0	0.94	0.32
*Keeping an eye*	8 (6–9)	7–9, 65.0 1–3, 0	8 (7–9)	7–9, 77.0 1–3, 0	0.77	0.24
*Managing challenging behaviour*	9 (5.5–9)	7–9, 70.0 1–3, 20.0	9 (7–9)	7–9, 77.0 1–3, 15.0	0.85	0.34
*Help provided in the home, for example, sitting/befriending service (n=19)*	9 (5–9)	7–9, 68.4 1–3, 5.3	9 (8.5–9)	7–9, 83.0 1–3, 8.0	0.89	0.08
Help provided in a residential setting e.g. residential/nursing home or hospital (n=18)	6.5 (5-9)	7–9, 50.0 1–3, 16.7	7 (6-9)	7–9, 64.0 1–3, 9.0	0.97	0.92
*Carer’s assessment by local social services or health authority (n=19)*	7 (5-9)	7–9, 68.4 1–3, 10.5	7 (6.5–8.5)	7–9, 75.0 1–3, 8.0	0.92	0.77

Items in bold reached criteria for inclusion in both round 1 and round 2, items in italics reached criteria for inclusion in round 2.

GP, general practitioner; ICC, intraclass correlation coefficient; NMLD, non-memory led dementia.

**Table 2:** * *New resource use items added in free text of modified Delphi study (scores reported in online supplemental material)

Complementary therapies	Healthcare	Productivity
Private therapy	Podiatry	Adjustments at work around caring
Counselling	Occupational therapy	
Massage	Neurophysiotherapy	**Out-of-pocket costs**
Mindfulness	Speech and language therapy	Transport costs
Acupuncture		Other carer responsibilities, for example, childcare
Yoga	**Support services**	Home maintenance
Reflexology	Peer support	Lasting power of attorney
Aromatherapy	Young carer support	Costs related to incontinence
Relaxation therapy	Carers emergency support	Additional heating requirements
Reiki	Family support	
Pilates	Care training	
Spa	Signposting to services	
Music therapy		
Art therapy		
Aqua therapy		
Social activities—music, dance, painting		

Face-to-face general practitioner (GP) appointments were highly rated and met all conditions to include, whereas telephone and online appointments were not highly rated. Given changes in the way GP surgeries operate, with a greater emphasis on telephone appointments, and if circumstances require a greater use of online services, it was decided to keep all options for appointment delivery included. The final questions on GP and practice nurse appointments do not distinguish between face-to-face, telephone or online, capturing all appointments without requiring additional questions.

The RDS groups and 1:1 calls were highly rated, with other support groups (peer, young carer, emergency and family support) added by a number of participants. An additional item on other support was included to capture support groups other than RDS.

Of the new items suggested in round 1, speech and language therapy and lasting power of attorney were considered very important in round 2. On discussion with the RD-TALK research team, it was decided these items were likely to be for the person with rare dementia and not for the carer and so were excluded from the final RUM.

The greatest consensus was in the productivity and carer tasks. In both round 1 and 2, time off work to attend appointments with a person with NMLD or taking sick leave due to caring responsibilities met all criteria for inclusion. All caring tasks were highly rated and met all criteria for inclusion by round 2.

Carers tended to give lower ratings for healthcare items than HCPs, and only RDS direct support was rated as very important. HCPs consistently rated healthcare items as higher importance—community mental health services, GP and practice nurse appointments, and psychological interventions. RDS groups and direct support were all rated very important and met the criteria to keep. A similar pattern was seen in the productivity and personal cost items, with time off work considered very important by all HCP respondents, meeting the criteria to keep in both rounds. Of the 10 caring tasks, only physical help was not considered very important by HCPs to include in the first round. By round 2, all caring tasks were rated as very important, with 100% of respondents scoring these between 7 and 9. The carers only rated three care tasks as very important and met the criteria to keep: help dealing with care services and benefits, other paperwork or financial matters and giving medicines. HCPs also highly rated help provided in the home and the carer’s assessment, but help provided in a residential setting was not considered to be important; the carers’ scores did not reach the criteria for very important for any of these items.

### Pilot study validation

The RUM was revised with the results of the modified Delphi study and delivered in the first 3 months of the BELIDE trial. There were 13 participants recruited in this time, of which 11 completed the economic outcome measures including the RUM (table 3). Response rates were good for healthcare resources with limited missing data; personal costs were not as well reported, which may be due to non-response if these items were not relevant to the participant. There were some items with no recorded contacts, such as community mental health services. On discussion with the research team, it was decided not to remove them based on the small sample size. Some items were reported twice due to confusion over provision of services, either NHS or private care, and were reworded for the final RUM to avoid double reporting.

**Table 3:** Results of pilot trial

Primary and community healthcare (n=11)	Number of participants who used this service	Mean number of appointments
GP	2	2
Practice nurse	1	1
Pharmacy	1	4
Psychological therapy (n=11)	Number of participants who used this service	Mean number of appointments
Psychological therapy in-person, online or telephone	1	16
Community mental health appointment in a clinic, online or at home	0	0
Rare Dementia Support group	1	1
Rare Dementia Support 1:1 call	1	1
Other support group	0	0
Private healthcare (n=11)	Number of participants who used this service	Mean number of appointments
Psychotherapy	2	12
Psychiatrist	1	2
GP	1	2
Complementary therapy	0	0
Employment and benefits	Yes responses	
In receipt of carer allowance (n=11)	0	
Early retirement (n=11)	1	
Employer has made adjustments (n=9)	2	
Time off for healthcare appointments (n=8)	2	
Sick leave (n=8)	2	
Personal care	Yes responses	
Personal care (n=11)	2	
Physical help (n=11)	1	
Help dealing with care service and benefits (n=11)	9	
Help with other paperwork or financial matters (n=11)	10	
Other practical help (n=11)	9	
Keeping him/her company (n=9)	7	
Taking him/her out (n=11)	9	
Giving medicines (n=11)	4	
Keeping an eye on him/her to see he/she is all right (n=10)	8	
Managing challenging behaviour (n=11)	1	
Help and support	Yes responses	
Respite care (n=11)	3	
Carers assessment (n=3)	1	
Home help—private care (n=3)	2	
Home help—family (n=3)	2	
Home help—local authority (n=3)	0	
Home help—charity (n=3)	0	
Other care	0	
Transport (n=11)	Yes responses	
Needed transport for healthcare appointments	5	
Own transport	5	
Taxis	1	
Public transport	2	
NHS ambulance or patient transport service	0	

GP, general practitioner; NHS, National Health Service.

There were GP, practice nurse and pharmacy visits reported, and respondents used private care in addition to NHS care. Only the RDS group was reported as a support group. The employment and benefits questions were all completed, with respondents reporting adjustments made by employers for their caring responsibilities, time off for healthcare appointments and needing to take sick leave. The carer tasks were all completed, the majority of respondents reported help dealing with care services and benefits, other paperwork or financial matters and other practical help, with far fewer respondents reporting personal care, physical help or managing challenging behaviour. These responses may reflect the stage of illness of the person with NMLD and associated symptoms in the small number of participants included. Respite care was required by a small number of respondents, and this was provided by private companies as well as friends and family.

When asking about medications, a number of medications did not appear relevant to caring responsibilities, although it is difficult to make this distinction. It was decided to include a second question on any other medication, and researchers will select which medications are relevant to caregiving. The RUM was updated with these agreed changes and is now being used in the main BELIDE trial.

## Discussion

We worked with carers and HCPs to develop a RUM for unpaid carers of people living with NMLD. The productivity and carer task items were rated as very important and met the inclusion criteria to keep. For healthcare, GP appointments, psychological interventions and RDS support were highly rated. The HCPs consistently rated items higher than the carers, with carers only rating RDS direct support as very important and meeting the criteria for inclusion in the first round from the healthcare items. Whereas the HCPs rated all caring tasks except for physical help as very important, the carers rated only three care tasks as very important, help dealing with care services and benefits, other paperwork and financial matters and giving medicines. This may reflect the population with NMLD who are younger and less likely to require physical support. Defining carer tasks can be subjective, as some people will identify tasks as normal interactions with a family member or friend requiring care. Collecting resource use related to care tasks is important for this population, and distinguishing between the tasks provides a valuable picture of care for people with NMLD, which is often unpaid, and can guide future research questions and design and ensures we are providing a complete picture of the costs related to being an unpaid carer. It is interesting to note that HCPs rated several healthcare items as very important compared with lower ratings from carers, such as community mental health services and psychological therapy. This may be due to difficulties for carers in accessing these services, or potentially not knowing that these services are available to them.

The RUM developed through the interviews and modified Delphi method was tested in an embedded pilot study for BELIDE to determine if the RUM was written in a clear and understandable way to allow accurate responses. Response rates were good for healthcare resources with limited missing data, personal costs were not as well reported, which may be due to non-response if these items were not relevant to the participant. There were some items with no recorded contacts which were discussed with the research team and due to the short time horizon of the pilot study, it was decided they should remain in the RUM as these items were identified in the interviews with PPIs and HCPs. Some items were reported twice due to confusion over provision of services, either NHS or private care; these questions were reworded to avoid double reporting.

The main limitation was the small number of carer participants as they are best placed to understand the resource use implications of caring, and this reduces diversity of opinions. Carers of people with Lewy body dementia were not included, which is a limitation as memory is typically less affected than other abilities early in the course of Lewy body dementia. The measure was designed for carers of people with dementia types where specific cognitive functions other than memory are the initial functions affected, in part due to the context of the trials in which the RUM is intended to be used.

A pragmatic approach was used with convenience sampling to use existing networks to recruit study participants, and while this may not capture a fully representative population, the Delphi Survey was built on extensive qualitative work and literature reviews and was piloted in an initial stage of the full trial. The Delphi method facilitates the gathering of a group consensus and further identifies areas where diversity of opinion exists^20^ and provides a practical way of developing a RUM within the set-up and internal pilot phase of a full RCT. Recruitment in rare diseases has additional problems due to the small populations and with specialised care centres it can be difficult to collect geographically representative data.^24^ Using social media and collaborating with patient groups has been found to be an effective method for attracting geographically representative samples.^24^

A difficulty for this population is making the distinction between healthcare items related specifically to caring for someone with NMLD and not to general health issues or to the health of the person they are caring for. This was seen in some of the additional items suggested, such as speech and language therapy. The focus of the RUM has been made clear in each question, although this is likely to remain an issue in this population where a carer is closely involved in accessing healthcare for the person with NMLD. The methodology for inclusion of carer outcomes into economic evaluation is not clear. A cross-sectional UK-wide survey of people with young-onset dementia reported that over one-third of family carers were in paid employment and more than half had given up work to provide care.^45^ Almost 60% of family members reported spending more than 5 hours per day supervising/helping the person with young-onset dementia, and around 70% of family members had neither attended a carers’ group nor reported using respite care.^45^ A high rate of acute medical care has been reported for caregivers, related to depression, care recipient behaviour problems and functioning, with increasing dependence of individuals with dementia associated with increased caregiver healthcare use.^46^

Valuing unpaid care for economic evaluation can be complicated with problems regarding validity and reliability in time estimates.^47^ There is currently no consensus on the best method or how to collect data on caregiving time and tasks to inform calculations. An evaluation of a specialist nursing service with a specific focus on supporting carers of people with dementia obtained information on care using a list of 10 different tasks taken from the 2009 ‘survey of carers in households’.^43 44^ By using this comprehensive list, developed for UK policy reports, we can develop a comprehensive picture and related costs of the care tasks carried out by people caring for someone with NMLD.

Evidence-based interventions are needed for unpaid carers that provide good value for money to budget holders. By working with carers of people with NMLD to develop a RUM for the BELIDE trial, we have been able to ensure that the most relevant resource items are included for decision-makers, HCPs working with carers and, most importantly, the carers themselves. This preparatory work in developing a validated RUM enables us to better describe health and social care resource use, as well as personal costs and time for unpaid care to develop a comprehensive picture of resource burden within the NHS, personal social services and to wider society related to unpaid care which will inform future economic evaluations.

## Conclusion

Given the considerable impact on well-being and productivity of informal carers, interventions are needed to support people providing care. To determine the cost-effectiveness of interventions requires evaluation with accurate measurement of resource use and associated costs, incorporating the key cost drivers, but without being a burden to research participants. Informal carers and HCPs working with people with NMLD are the experts in the resource impact to the NHS and wider society of caring for someone with dementia. By working with these groups, we hope to have developed a comprehensive, valid, RUM for informal carers of people with NMLD.

## Supplementary material

10.1136/bmjopen-2025-110399 online supplemental file 1

10.1136/bmjopen-2025-110399 online supplemental file 2

10.1136/bmjopen-2025-110399 online supplemental file 3

## Data Availability

All data relevant to the study are included in the article or uploaded as supplementary information.
