# Supplementary material for: Development of a resource use measure to capture costs related to unpaid care for people living with non-memory led dementia: a modified Delphi study
Source: BMJ Open. 2026 Feb 12;16(2):e110399. doi: 10.1136/bmjopen-2025-110399 (PMC12911721; doi:10.1136/bmjopen-2025-110399)
Supplement: online supplemental file 1 [file bmjopen-16-2-s001.docx]

### PRISMA – people with dementia and carers

**Identification of studies via databases and registers**

Records removed *before screening*:

Duplicate records removed (n = 7,146)

15,876 Records identified from:

MEDLINE

CINAHL

PsychInfo

PubMed

Cochrane

NHSEED

INAHTA

**Identification**

Records excluded

(n =8,681)

Records screened

(n = 8,730)

**Screening**

Reports not retrieved

(n = 937)

Reports sought for retrieval

(n = 1025)

Reports assessed for eligibility

(n =88 )

Studies included in review

(n =15 )

**Included**

### Modified Delphi Questionnaire Round 1

**Help us to understand the costs of caring for a loved one with rare dementia for our work on online support programmes.**

**THE BACKGROUND**

Healthcare commissioners are unlikely to adopt new support programmes for people caring for someone with rare dementia unless they are good value for money. This means researchers need to ask everyone who tests our new programmes what costs they have associated with being a **carer for someone with a rare type of dementia** (Primary Progressive Aphasia (PPA), Posterior cortical atrophy (PCA), behavioural variant frontotemporal dementia (bvFTD)). Then, researchers will see what these new support programmes do to alleviate these costs.

**THE PROBLEM**

The problem is that the current ways of measuring value for money don’t capture many of the services that you, specifically as a carer for someone with rare dementia, may be using. So, we are **making a new measure of costs that specifically reflect your experiences of caring for someone living with rare dementia.** We will use this measure to test our new online programmes made for you. This new measure would be the first of its kind.

**HOW YOU CAN HELP**

To make this new measure, we first need your help to tell us which healthcare resources (for example GP appointments, counselling) that you have used related to caring for someone with rare dementia and wider resources that you could envisage yourself using (for example time off work, transport to appointments).

Specifically, we want to learn which of these you think are the most IMPORTANT to be included in this new cost measure.

**WHAT DO I HAVE TO DO?**

We are running a process called a Delphi study to create this new measure of rare dementia costs. Using this process involves asking you to complete up to three rounds of surveys until a consensus is reached on what needs to be included in the measure.

**Instructions for completing the process:**

- This survey will be one of a ****maximum of three**** we will ask you to complete for this research activity. They will get shorter each time.
- **Round 1:** This first survey asks you to rate the following 32 resource items on a scale of 1-9. ‘**1’ represents an item that is NOT IMPORTANT**, ‘**5’ represents an item that is IMPORTANT** and ‘**9’ represents an item that is VERY IMPORTANT** and should be included in the shortest version of the new cost measure. At the end of each section, there is opportunity to provide comments on your choice of items. At the end of the survey, you can add any additional items that are VERY IMPORTANT that you think we have missed, and should be included in the new cost measure. The survey should take no longer than 25 minutes to complete.
- We will use the responses from the first round to rank all the resource items and the overall rankings will be used to re-write the survey.
- **Round 2:** We will then ask you to re-rate the shorter list of items, which will include the overall rankings of the items from the first round, as well as any new items suggested.
- If consensus is reached on the items after this second round we will stop data collection,.
- **Round 3 (if needed):** If consensus is not reached, we will ask you to review and rate a third, and final list of items.

This Delphi consensus survey is part of the RDS Impact Study, ethical approval for which has been granted by the UCL Research Ethics Committee. You can read the full information sheet for the study by clicking [here](https://drive.google.com/file/d/1tdlgt-M1nKitNvToNd4qIoOmG8NtEkvC/view?usp=sharing).

Please tick the box below to indicate that you have read and understood the information provided to you about this study, and you agree to proceed to complete this online questionnaire.

Yes, I agree to proceed

No, I do not agree to proceed (skips to end of survey if selected)

Please tell us who is completing this questionnaire:

First name

Last name

Email address

**We would be grateful if you could complete all sections of the survey.**

## ***Section 1: Primary/Community Care***

Please rate the importance of the following resource items as a carer of someone with rare dementia (not for the person with dementia (e.g. what is critically important to include in the new cost measure). Please consider if you have used these services, or if you are aware they are available and they are something you, or someone else in a similar position, would be likely to use.

| **Item** | **Category** | **Resource Item** | **Rating of importance** | | | | | | | | |
| --- | --- | --- | --- | --- | --- | --- | --- | --- | --- | --- | --- |
| 1 | GP | Face-to-face | 1 | 2 | 3 | 4 | 5 | 6 | 7 | 8 | 9 |
| 2 |  | Online/telephone | 1 | 2 | 3 | 4 | 5 | 6 | 7 | 8 | 9 |
| 3 | Practice nurse | Face-to-face | 1 | 2 | 3 | 4 | 5 | 6 | 7 | 8 | 9 |
| 4 |  | Online/telephone | 1 | 2 | 3 | 4 | 5 | 6 | 7 | 8 | 9 |
| 5 | Community mental health team | Face-to-face | 1 | 2 | 3 | 4 | 5 | 6 | 7 | 8 | 9 |
| 6 |  | Online/telephone | 1 | 2 | 3 | 4 | 5 | 6 | 7 | 8 | 9 |
| 7 | Psychological intervention **online/computer only** | NHS talking therapy, Improving Access to Psychological Therapies (IAPT), iCOPE. | 1 | 2 | 3 | 4 | 5 | 6 | 7 | 8 | 9 |
| 8 | Psychological intervention **face-to-face - 1 to 6 sessions** | NHS talking therapy, Improving Access to Psychological Therapies (IAPT), iCOPE. | 1 | 2 | 3 | 4 | 5 | 6 | 7 | 8 | 9 |
| 9 | Psychological intervention **face-to-face** **– 7 or more sessions** | NHS talking therapy, Improving Access to Psychological Therapies (IAPT), iCOPE. | 1 | 2 | 3 | 4 | 5 | 6 | 7 | 8 | 9 |
| 10 | Pharmacy | Face-to-face | 1 | 2 | 3 | 4 | 5 | 6 | 7 | 8 | 9 |
| 11 | Support group | Rare Dementia Support groups |  |  |  |  |  |  |  |  |  |
| 12 | Specialist support | 1:1 direct support team call from RDS | 1 | 2 | 3 | 4 | 5 | 6 | 7 | 8 | 9 |
| **Please make any comments that you would like to make regarding the items in this section** | |  | | | | | | | | | |

## ***Section 2: Private healthcare costs***

Are there any therapies that you use, or you think other carers would use, that you pay for yourself, or through an employer’s scheme, or are provided by a charity? Please rate the importance of the following resource items as a carer of a person with rare dementia (e.g. what is critically important to include in the new cost measure). Please include psychological therapy, counselling, and complementary therapies, such as reiki, aromatherapy, massage therapy etc..

| **Item** | **Category** | **Resource Item** | **Rating of importance** | | | | | | | |  |
| --- | --- | --- | --- | --- | --- | --- | --- | --- | --- | --- | --- |
| 12 | Private healthcare | Private Healthcare (please list):  1.  2.  3.  4.  5.  6.  7.  8. | 1 | 2 | 3 | 4 | 5 | 6 | 7 | 8 | 9 |
| **Please make any comments that you would like to make regarding the items in this section** | |  | | | | | | | | | |

## ***Section 3: Costs of Travel and costs related to employment.***

Are there any costs for travelling to appointments or taking time off work, related to being a carer? Please rate the importance of the following resource item as a carer of a person with rare dementia (e.g. what is critically important to include in the new cost measure).

| **Item** | **Category** | **Resource Item** | **Rating of importance** | | | | | | | | |
| --- | --- | --- | --- | --- | --- | --- | --- | --- | --- | --- | --- |
| 14 | Time off work | Time off work to attend appointments with a person with rare dementia, paid and unpaid | 1 | 2 | 3 | 4 | 5 | 6 | 7 | 8 | 9 |
| 15 |  | Time off work/sick leave, paid and unpaid due to caring for a person with rare dementia | 1 | 2 | 3 | 4 | 5 | 6 | 7 | 8 | 9 |
| 16 | Benefits | Carer in receipt of allowance | 1 | 2 | 3 | 4 | 5 | 6 | 7 | 8 | 9 |
| 17 | Retirement | Early retirement due to caring for someone with rare dementia | 1 | 2 | 3 | 4 | 5 | 6 | 7 | 8 | 9 |
| **Please make any comments that you would like to make regarding the items in this section** | |  | | | | | | | | | |

## ***Section 4: Time related to caring for someone with rare dementia***

The following items will help us understand what is involved in caring for someone with rare dementia. Please rate the importance of the following activities (e.g. what is critically important to include in the new cost measure).

| **Item** | **Category** | **Resource Item** | **Rating of importance** | | | | | | | | |
| --- | --- | --- | --- | --- | --- | --- | --- | --- | --- | --- | --- |
| 18 | Personal care | e.g. dressing, bathing, washing, shaving, cutting nails, feeding, using the toilet | 1 | 2 | 3 | 4 | 5 | 6 | 7 | 8 | 9 |
| 19 | Physical help | e.g. with walking, getting up and down stairs, getting into and out of bed | 1 | 2 | 3 | 4 | 5 | 6 | 7 | 8 | 9 |
| 20 | Helping with dealing with care services and benefits | e.g. making appointments and phone calls, filling in forms | 1 | 2 | 3 | 4 | 5 | 6 | 7 | 8 | 9 |
| 21 | Helping with other paperwork or financial matters | e.g. writing letters, filling in forms, dealing with bills. | 1 | 2 | 3 | 4 | 5 | 6 | 7 | 8 | 9 |
| 22 | Other practical help | e.g. preparing meals, doing his/her shopping, laundry, housework, household repairs | 1 | 2 | 3 | 4 | 5 | 6 | 7 | 8 | 9 |
| 23 | Keeping him/her company | Visiting ,sitting with, reading to, talking to | 1 | 2 | 3 | 4 | 5 | 6 | 7 | 8 | 9 |
| 24 | Taking him/her out | e.g. for a walk or drive, taking to see friends or relatives | 1 | 2 | 3 | 4 | 5 | 6 | 7 | 8 | 9 |
| 25 | Giving medicines | e.g. making sure he/she takes pills, giving injections | 1 | 2 | 3 | 4 | 5 | 6 | 7 | 8 | 9 |
| 26 | Keeping an eye on him/her to see he/she is all right |  | 1 | 2 | 3 | 4 | 5 | 6 | 7 | 8 | 9 |
| 27 | Managing challenging behaviour |  |  |  |  |  |  |  |  |  |  |
| **Please make any comments that you would like to make regarding the items in this section** | |  | | | | | | | | | |

## ***Section 5: Help and support***

Is there any external help and support that you can access either through social services, the NHS, charities, or you pay for yourself? Please rate the importance of the following (e.g. what is critically important to include in the new cost measure).

| **Item** | **Category** | **Resource Item** | **Rating of importance** | | | | | | | | |
| --- | --- | --- | --- | --- | --- | --- | --- | --- | --- | --- | --- |
| 28 | Help provided in the home | e.g. sitting/befriending service | 1 | 2 | 3 | 4 | 5 | 6 | 7 | 8 | 9 |
| 29 | Help provided in a residential setting | e.g. residential/nursing home or hospital | 1 | 2 | 3 | 4 | 5 | 6 | 7 | 8 | 9 |
| 30 | Carer’s assessment of your own needs | By local social services or health authority | 1 | 2 | 3 | 4 | 5 | 6 | 7 | 8 | 9 |
| **Please make any comments that you would like to make regarding the items in this section** | |  | | | | | | | | | |

## ***Section 6: Additional Items***

**Please list any other items that are CRITICAL to include in the new cost measure:**

| Item | Any additional comment |
| --- | --- |
|  |  |
|  |  |
|  |  |

**OR click the arrow to complete the survey.**

Thank you for taking part in Round 1 of the Delphi consensus survey.

****What happens next?****
We will collate your answers with the responses of other people caring for someone with a rare dementia, and be back in touch within 10-12 days to send you the updated measure to review and comment on.

****I have a question:****
If you have any questions about taking part in this consensus survey, please email [research@raredementiasupport.org](mailto:research@raredementiasupport.org?subject=Delphi%20consensus%20study%20-%20FAO%20Dr%20Emilie%20Brotherhood).

### Modified Delphi – results for new items suggested in round 1

Blue = Percentage agreement = keep; IQR ≤2

Green = Percentage agreement = keep; IQR >2

Yellow = Percentage agreement = remove; IQR>2

| **Item** | **Mean** | **SD** | **Median** | **IQR** | **Range** | **Percentage agreement** |
| --- | --- | --- | --- | --- | --- | --- |
| Counselling | 7.3 | 1.7 | 7.5 | 6 – 9 | 5 - 9 | 7–9 60%  1-3 0% |
| Massage | 4.3 | 2.4 | 4 | 3 – 5 | 1 - 9 | 7–9 20%  1-3 40% |
| Mindfulness | 5.6 | 2.4 | 5.5 | 5 – 7 | 1 - 9 | 7–9 40%  1-3 20% |
| Acupuncture | 2.9 | 2.0 | 2.5 | 1 – 5 | 1 - 6 | 7–9 0%  1-3 60% |
| Yoga | 3.5 | 2.7 | 2.5 | 1 – 5 | 1 - 9 | 7–9 10%  1-3 60% |
| Reflexology | 3 | 1.9 | 3 | 1 – 5 | 1 - 5 | 7–9 0%  1-3 50% |
| Aromatherapy | 2.9 | 1.9 | 3 | 1 – 5 | 1 - 5 | 7–9 0%  1-3 50% |
| Relaxation therapy | 4.6 | 2.6 | 5 | 2 – 6 | 1 - 9 | 7–9 20%  1-3 40% |
| Reiki | 2.8 | 2.3 | 1.5 | 1 – 5 | 1 - 7 | 7–9 10%  1-3 60% |
| Pilates | 2.8 | 1.8 | 2.5 | 1 – 5 | 1 - 5 | 7–9 0%  1-3 60% |
| Spa | 1.9 | 1.7 | 1 | 1 – 2 | 1 - 6 | 7–9 0%  1-3 89% |
| Music therapy | 5.8 | 2.4 | 6 | 5 – 7 | 1 - 9 | 7–9 45%  1-3 18% |
| Art therapy | 4.7 | 2.7 | 5 | 3 – 7 | 1 - 9 | 7–9 30%  1-3 40% |
| Podiatry | 4.6 | 3.1 | 5 | 1 – 7 | 1 - 9 | 7–9 33%  1-3 33% |
| Relate | 4.5 | 3.1 | 4.5 | 1 – 7 | 1 - 9 | 7–9 30%  1-3 40% |
| Aqua therapy | 2.8 | 2.6 | 1.5 | 1 – 4 | 1 - 9 | 7–9 10%  1-3 70% |
| Occupational therapy | 6.1 | 3.0 | 7 | 4 – 9 | 1 - 9 | 7–9 60%  1-3 20% |
| Exercises | 5.7 | 2.3 | 5.5 | 5 – 8 | 1 - 9 | 7–9 30%  1-3 10% |
| Neurophysiotherapy | 4.7 | 2.6 | 5 | 3 – 6 | 1 - 9 | 7–9 20%  1-3 30% |
| Speech and language therapy | 7.5 | 2.6 | 8.5 | 8 – 9 | 1 - 9 | 7–9 80%  1-3 10% |
| Transport costs | 5.8 | 3.0 | 5 | 4 – 9 | 1 - 9 | 7–9 38%  1-3 23% |
| Social activities – music, dance, painting | 7.2 | 2.4 | 8 | 6 – 9 | 1 - 9 | 7–9 75%  1-3 8% |
| Peer support | 7.2 | 2.3 | 8 | 7 – 9 | 1 - 9 | 7–9 77%  1-3 8% |
| Language/communication support | 6.3 | 3.0 | 7.5 | 5 – 9 | 1 - 9 | 7–9 58%  1-3 17% |
| Young carer support | 6.3 | 3.5 | 8 | 2.5 – 9 | 1 - 9 | 7–9 67%  1-3 25% |
| Carers emergency support | 7.3 | 3.1 | 9 | 6.5 – 9 | 1 - 9 | 7–9 75%  1-3 17% |
| Family support | 7.5 | 1.9 | 9 | 7 – 9 | 4 - 9 | 7–9 77%  1-3 0% |
| Free care training | 5.8 | 2.6 | 5 | 5 – 8.5 | 1 - 9 | 7–9 33%  1-3 17% |
| Signposting to services | 7 | 2.4 | 7 | 6 – 9 | 1 - 9 | 7–9 62%  1-3 8% |
| Other carer responsibilities – e.g. childcare | 5.3 | 3.5 | 5.5 | 1 – 9 | 1 - 9 | 7–9 42%  1-3 33% |
| Home maintenance | 5.5 | 2.7 | 5 | 4 – 8 | 1 - 9 | 7–9 31%  1-3 15% |
| Lasting power of attorney | 8.2 | 1.3 | 9 | 8 - 9 | 5 - 9 | 7–9 85%  1-3 0% |
| Costs related to incontinency | 6.8 | 2.5 | 7 | 5 – 9 | 1 - 9 | 7–9 54%  1-3 8% |
| Additional heating requirements | 6.6 | 2.5 | 6.5 | 5 – 9 | 1 - 9 | 7–9 50%  1-3 8% |
| Technology for speech activation | 5.4 | 2.7 | 5 | 5 – 7.5 | 1 - 9 | 7–9 25%  1-3 17% |
| Yearly carer health checks by GP | 7.2 | 2.2 | 8 | 5 – 9 | 3 - 9 | 7–9 64%  1-3 9% |
| Wellbeing assessment | 6 | 3.0 | 6 | 5 – 9 | 1 - 9 | 7–9 45%  1-3 18% |
| Costs pre-diagnosis | 5.3 | 2.4 | 5 | 4 – 7 | 1 - 9 | 7–9 27%  1-3 18% |

### RD-talk Final Resource Use Measure – carers

**Please complete these questions for your own healthcare appointments related to being a carer and not appointments for the person you care for. Please tick ‘yes’ or ‘no’ for each item. If you answer ‘yes’, please fill in the additional questions related to that item.**

##

## *Primary Care and Community Care*

1. **In the last 8 weeks, have you visited or spoken to your GP, a practice nurse or a pharmacist about your health and wellbeing in relation to your caring responsibilities?**

|  | **Please select yes for any item which applies** | **Number of appointments/visits/ phone calls in the last 8 weeks** |
| --- | --- | --- |
| GP appointment (NHS), either attending the GP surgery or by phone/online | Yes  No |  |
| Practice nurse appointment (NHS), either in person or by phone/online | Yes  No |  |
| Pharmacy | Yes  No |  |

## *NHS Psychological Therapy, Community Mental Health Care and Support Groups*

1. **In the last 8 weeks, have you had any psychological intervention sessions, such as NHS Talking Therapies (previously called Improving Access to Psychological Therapies (IAPT) or iCOPE), or had an appointment or home visit with the community mental health team in relation to your role as a carer?**

|  | **Please select yes for any item which applies** | **Number of sessions/visits/calls** | **On average, how many minutes was each session/visit/call?** |
| --- | --- | --- | --- |
| NHS Psychological therapy either in-person or online or on the telephone | Yes    No |  |  |
| Community NHS Mental Health team appointment in a clinic or online | Yes  No |  |  |
| Community NHS Mental Health team home visit | Yes  No |  |  |
| Rare Dementia Support group | Yes  No |  |  |
| Rare Dementia Support 1:1 call | Yes  No |  |  |
| Other support group, for example young carer support | Yes  No |  |  |

## *Private care*

1. **In the last 8 weeks, have you, private insurance, or a charity paid for any health care or complementary therapies related to your role as a carer?**

**Please complete these questions for your own appointments related to being a carer and not appointments for the person you care for.**

|  |  |  |
| --- | --- | --- |

| **Private healthcare -**  **Please write the healthcare provided for example psychological therapy, occupational therapy** | **Please select yes for any item which applies** | **Number of appointments/sessions** |
| --- | --- | --- |
| 1. | Yes  No |  |
| 2. | Yes  No |  |
| 3. | Yes  No |  |

| **Private Complementary therapy –for example; yoga, reiki, aromatherapy, acupuncture** | **Please select yes for any item which applies** | **Number of appointments/sessions** |
| --- | --- | --- |
| 1. | Yes  No |  |
| 2. | Yes  No |  |
| 3. | Yes  No |  |

## *Medicines*

1. **In the last 8 weeks, have you taken any medicines, either prescribed or over the counter?**

**As with the healthcare appointments, please just include medications you take yourself and not those taken by the person you care for.**

| **Medicines taken in relation to being a carer**      **Name:** | **Dose per day** | **How long in months have you been using this medicine?** | **Please select if this medication was prescribed or purchased over the counter?** |
| --- | --- | --- | --- |
|  |  |  | Prescription  Over the counter |
|  |  |  | Prescription  Over the counter |
|  |  |  | Prescription  Over the counter |

| **Medicines taken NOT in relation to being a carer**        **Name:** | **Dose per day** | **How long in months have you been using this medicine?** | **Please select if this medication was prescribed or purchased over the counter?** |
| --- | --- | --- | --- |
|  |  |  | Prescription  Over the counter |
|  |  |  | Prescription  Over the counter |
|  |  |  | Prescription  Over the counter |

### Devices and Equipment

1. **In the last 8 weeks, have you bought or been provided by the NHS, a charity or the local authority, any devices, technology or other equipment related to caring for someone with dementia?**

| **Device/equipment (please write the type of device)** | **Please select who provided these** |
| --- | --- |
|  | Own purchase    NHS    Local Authority    Charity |
|  | Own purchase    NHS    Local Authority    Charity |
|  | Own purchase    NHS    Local Authority    Charity |

## *Employment and benefits*

Do you receive carer’s allowance? Yes No

| Have you taken early retirement due to your caring responsibilities? Yes No | | |
| --- | --- | --- |
| Has your employer made adjustments at work around your caring responsibilities?  Yes  No  I’m self employed  I’m not working | | |
|  | **Please select yes for any item which applies** | **Number of whole working days taken off** |
| Have you taken time off work, paid or unpaid, to attend appointments for your own health and wellbeing in the last 8 weeks? | Yes  No |  |
| Have you taken sick leave for your own health and wellbeing due to your caring responsibilities in the last 8 weeks? | Yes  No |  |

## *Personal Care*

1. **Please tell us how much time you have spent providing care for the person with rare dementia in the last 8 weeks.**

|  | **Please select yes for any item which applies** | **How long in months have you provided this care?** | **On average, how many hours per week do you provide this care?** |
| --- | --- | --- | --- |
| Personal care for example dressing, bathing, washing, shaving, cutting nails, feeding, using the toilet | Yes  No |  |  |
| Physical help for example with walking, getting up and down stairs, getting into and out of bed | Yes  No |  |  |
| Helping with dealing with care services and benefits for example making appointments and phone calls, filling in forms | Yes  No |  |  |
| Helping with other paperwork or financial matters for example writing letters, filling in forms, dealing with bills | Yes  No |  |  |
| Other practical help for example preparing meals, doing their shopping, laundry, housework, household repairs | Yes  No |  |  |
| Keeping them company for example visiting, sitting with, reading to, talking to | Yes  No |  |  |
| Taking them out for example for a walk or drive, taking to see friends or relatives, social activities, accompanying to appointments | Yes  No |  |  |
| Giving medicines for example making sure them takes pills, giving injections | Yes  No |  |  |
| Keeping any eye on them to see they are all right | Yes  No |  |  |
| Managing challenging behaviour | Yes  No |  |  |

## *Help and support*

1. **Have you needed support in the last 8 weeks to provide care for someone with dementia, for example respite care, or additional childcare?**

This can be provided by friends or family, your local authority, a charity or a private company.

| Have you had a carer’s assessment? (an evaluation by the local authority to understand what support you need) Yes No | | | | |
| --- | --- | --- | --- | --- |
|  | **Please select yes for any item which applies** | **Who provided this help/support?** | **How long have you needed respite/ other care in months?** | **On average, how many hours of support per week were provided?** |
| Help provided in the home, for example, sitting/befriending service, respite care | Yes    No | Private company  Relatives/friends  Local authority  Charity |  |  |
| Other carer responsibilities, for example, have you needed additional childcare while you were caring for someone with rare dementia | Yes    No | Private company  Relatives/friends  Local authority  Charity |  |  |

## *Transport*

1. **Have you needed transport to go to healthcare appointments for yourself related to your caring responsibilities, or to accompany the person you care for to healthcare appointments in the last 8 weeks?**

**Please include own transport, public transport, taxis, NHS ambulance or patient transport service.**

|  | **Please select yes for any item which applies** | **On average, how many times per week did you use this transport to attend healthcare appointments?** | **Please can you indicate the cost per journey in pounds and pence** |
| --- | --- | --- | --- |
| Own transport | Yes    No |  |  |
| Taxis | Yes    No |  |  |
| Public transport | Yes    No |  |  |
|  |  | **Can you tell us how many times you used these transport services in the last 8 weeks?** | |
| NHS ambulance | Yes    No |  | |
| Patient transport service | Yes    No |  | |
