# Supplementary material for: Development of a resource use measure to capture costs related to unpaid care for people living with non-memory led dementia: a modified Delphi study
Source: BMJ Open. 2026 Feb 12;16(2):e110399. doi: 10.1136/bmjopen-2025-110399 (PMC12911721; doi:10.1136/bmjopen-2025-110399)
Supplement: online supplemental file 2 [file bmjopen-16-2-s002.docx]

## Conducting and Reporting of Delphi Studies (CREDES)

Jünger, S., Payne, S. A., Brine, J., Radbruch, L., & Brearley, S. G. (2017). [Rev. of Guidance on Conducting and REporting DElphi Studies (CREDES) in palliative care: Recommendations based on a methodological systematic review]. Palliative Medicine, 31(8), 684–706. <https://doi.org/10.1177/0269216317690685>

| **Rationale for the choice of the Delphi technique** |  |
| --- | --- |
| 1. *Justification*. The choice of the Delphi technique as a method of systematically collating expert consultation and building consensus needs to be well justified. When selecting the method to answer a particular research question, it is important to keep in mind its constructivist nature | Introduction, page 4-5, 2nd paragraph; Objectives, page 5; Stage Three: modified delphi study, page 6-7, 1^st^ paragraph. |
| **Planning and design** |  |
| 1. *Planning and process*. The Delphi technique is a flexible method and can be adjusted to the respective research aims and purposes. Any modifications should be justified by a rationale and be applied systematically and rigorously. | Methods – stage three, page 6-7 |
| 1. *Definition of consensus*. Unless not reasonable due to the explorative nature of the study, an a priori criterion for consensus should be defined. This includes a clear and transparent guide for action on (a) how to proceed with certain items or topics in the next survey round, (b) the required threshold to terminate the Delphi process and (c) procedures to be followed when consensus is (not) reached after one or more iterations. | Methods – data analysis, page 7 - 8 |
| **Study conduct** |  |
| 1. *Informational input*. All material provided to the expert panel at the outset of the project and throughout the Delphi process should be carefully reviewed and piloted in advance in order to examine the effect on experts’ judgements and to prevent bias. | Methods – stage two, page 6 |
| 1. *Prevention of bias*. Researchers need to take measures to avoid directly or indirectly influencing the experts’ judgements. If one or more members of the research team have a conflict of interest, entrusting an independent researcher with the main coordination of the Delphi study is advisable. | Methods – stage three, page 7, 4^th^ paragraph - ‘potential participants were provided with an invitation email... including a link to an online platform.’ The main researcher is not a part of Rare Dementia Support. |
| 1. *Interpretation and processing of results.* Consensus does not necessarily imply the ‘correct’ answer or judgement; (non)consensus and stable disagreement provide informative insights and highlight differences in perspectives concerning the topic in question. | Methods – data analysis, page 7-8, consensus and criteria for including or removing items stated. |
| 1. *External validation*. It is recommended to have the final draft of the resulting guidance on best practice in palliative care reviewed and approved by an external board or authority before publication and dissemination. | N/A |
| **Reporting** |  |
| 1. *Purpose and rationale*. The purpose of the study should be clearly defined and demonstrate the appropriateness of the use of the Delphi technique as a method to achieve the research aim. A rationale for the choice of the Delphi technique as the most suitable method needs to be provided. | Introduction, page 4-5, 2nd paragraph; Objectives, page 5; Stage Three: modified delphi study, page 6-7, 1^st^ paragraph. |
| 1. *Expert panel*. Criteria for the selection of experts and transparent information on recruitment of the expert panel, sociodemographic details including information on expertise regarding the topic in question, (non) response and response rates over the ongoing iterations should be reported. | Methods -stage three, page 6-7, 1^st^ and 2^nd^ paragraphs |
| 1. *Description of the methods*. The methods employed need to be comprehensible; this includes information on preparatory steps (How was available evidence on the topic in question synthesised?), piloting of material and survey instruments, design of the survey instrument(s), the number and design of survey rounds, methods of data analysis, processing and synthesis of experts’ responses to inform the subsequent survey round methodological decisions taken by the research team throughout the process. | Methods – stage one – literature search, page 6; stage two – initial discussions, page 6; stage three – modified delphi study, page 6-7; final stage pilot study, page 8-9. |
| 1. *Procedure*. Flow chart to illustrate the stages of the Delphi process, including a preparatory phase, the actual ‘Delphi rounds’, interim steps of data processing and analysis, and concluding steps. | Figure 1 |
| 1. *Definition and attainment of consensus*. It needs to be comprehensible to the reader how consensus was achieved throughout the process, including strategies to deal with non-consensus. | Methods – data analysis, page 7-8. |
| 1. *Results*. Reporting of results for each round separately is highly advisable in order to make the evolving of consensus over the rounds transparent. This includes such as deletion, addition or modification of survey items based on previous rounds. | Table 2, Results – modified delphi study pages 10 – 12, pilot study validation pages 14-16 |
| 1. *Discussion of limitations*. Reporting should include a critical reflection of potential limitations and their impacts of the resulting guidance. | Discussion, page 16-18 |
| 1. *Adequacy of conclusions*. The conclusions should adequately reflect the outcomes of the Delphi study with a view to the scope and applicability of the resulting practice guidance. | Conclusion , page 18-19 |
| 1. *Publication and dissemination*. The resulting guidance on good practice in palliative care should be clearly identifiable from the publication, including recommendations for transfer into practice and implementation. If the publication does not allow for a detailed presentation of either the resulting practice guidance or the methodological features of the applied Delphi technique, or both, reference to a more detailed presentation elsewhere should be made (e.g. availability of the full guideline from the authors or online; publication of a separate paper reporting on methodological details and particularities of the process (e.g. persistent disagreement and controversy on certain issues)). A dissemination plan should include endorsement of the guidance by professional associations and health care authorities to facilitate implementation | N/A |
