# Supplementary material for: Development of a resource use measure to capture costs related to unpaid care for people living with non-memory led dementia: a modified Delphi study
Source: BMJ Open. 2026 Feb 12;16(2):e110399. doi: 10.1136/bmjopen-2025-110399 (PMC12911721; doi:10.1136/bmjopen-2025-110399)
Supplement: online supplemental file 3 [file bmjopen-16-2-s003.pdf]

NIHR | National Institute for Health and Care Research

PROSPERO
International prospective register of systematic reviews

Methods for economic evaluation of in-person and digital interventions for genetic non-memory led dementias

Katherine Cullen, Deborah Fitzsimmons

Citation
Katherine Cullen, Deborah Fitzsimmons. Methods for economic evaluation of in-person and digital interventions for genetic non-memory led dementias. PROSPERO 2024 Available from https://www.crd.york.ac.uk/PROSPERO/view/CRD42022356943

REVIEW TITLE AND BASIC DETAILS

Review title
Methods for economic evaluation of in-person and digital interventions for genetic non-memory led dementias

Review objectives
1. What is the best practice for economic evaluation of in-person or digital interventions for people diagnosed with genetic non-memory led dementia (GNMLD)?
2. What is the best practice for economic evaluation of in-person or digital interventions for carers of people diagnosed with GNMLD?
3. What is the best practice for economic evaluation of in-person or digital interventions for people at risk of developing GNMLD?

Keywords
Analyses, Carers, Cost benefit, Cost effectiveness, Dementia

SEARCHING AND SCREENING

Searches
Sources:
MEDLINE, PubMed, the Cochrane Library, PsycINFO, CINAHL, NHSEED (up to 31st March 2015), INAHTA, and Google Scholar.
Restrictions:
1995 to present to reflect internet use and availability.
English language.

Evaluations will be included where a study population includes the population of interest.
Adults aged 18 years and older.
Additional search strategy information can be found in the attached PDF document (link provided below).

Study design
Full and partial published economic evaluations, methodological review papers and systematic reviews.

ELIGIBILITY CRITERIA

Condition or domain being studied
Dementia is a syndrome of progressive deterioration of cognitive function sufficient to impact on daily life and occupational functioning. Rarer, under-researched types of dementia that are either genetic (inherited from a parent) or non-memory led (initial difficulties with language, vision or personality changes, rather than memory) mostly affect people under 65 years, which can lead to delays in diagnosis as dementia is frequently thought of as a condition associated with old age. GNMLDs pose additional challenges due to the younger age at onset and unusual symptoms which can affect behaviour and social functioning, causing employment issues, and impacting on caring responsibilities.

Population
Non-memory led dementia/ genetic dementia/ semantic dementia/early onset dementia/ frontotemporal dementia/ Lewy body disease/ mild cognitive impairment/primary progressive aphasia/ posterior cortical atrophy/ Benson’s syndrome.
For carers, in addition to the terms above the following will also be considered: carer, caregiver, informal care, support, family (parent, mother, father, son, daughter, child, relative), spouse (husband, wife), neighbour.
For people at risk of developing GNLMD in addition to the population terms above the following will also be considered: Huntington’s disease, Spinocerebellar ataxia, breast cancer, ovarian cancer, colorectal cancer, prostate cancer, BRCA1, BRCA2, muscular dystrophy, haemochromatosis.

Intervention(s) or exposure(s)
In-person and digital therapy.
The interventions of interest are blended in-person/digital (e.g., on-line talking-groups, interactive websites, internet-based interventions), however, to identify all evidence related to assessing these interventions, all in-person and digital interventions, in any healthcare or social care context, will be included in the review. Examples of interventions include Improving Access to Psychological Therapies, cognitive simulation therapy, reality orientation, reminiscence therapy, psychological therapy, behavioural therapy, virtual-cognitive simulation therapy, talking therapy, word training, and function communication.

Comparator(s) or control(s)
Standard/usual care, active controls.

Context

OECD countries: Australia, Austria, Belgium, Canada, Chile, Colombia, Costa Rica, Czech Republic, Denmark, Estonia, Finland, France, Germany, Greece, Hungary, Iceland, Ireland, Israel, Italy, Japan, Korea, Latvia, Lithuania, Luxembourg, Mexico, Netherlands, New Zealand, Norway, Poland, Portugal, Slovak Republic, Slovenia, Spain, Sweden, Switzerland, Turkey, United Kingdom, United States.

OUTCOMES TO BE ANALYSED

Main outcomes
This review is required to understand the key parameters and methodological/analytical considerations to include in an economic evaluation to assess interventions for people diagnosed with GNMLD, their carers, and those at risk of developing GNMLD.
Main outcome - cost-effectiveness.

Measures of effect
Incremental cost-effectiveness or utility ratio.
Net monetary benefit.

Additional outcomes
Resource use and costs.
Quality adjusted life years.
Life years gained.
Perspective of evaluations.
Cost of illness.
Budget impact.

DATA COLLECTION PROCESS

Data extraction (selection and coding)
Data extraction will reflect the reporting standards as set out in the CHEERS checklist: Title, citation, country, source of funding, study population, setting and location, comparators, perspective, time horizon, discount rate, outcomes, resources and costs, currency including year, modelling methodology, analytics for transforming data, assumptions, heterogeneity, distributional effects, uncertainty, engagement with patients, parameters, main results, and limitations.

Risk of bias (quality) assessment
All full text economic evaluations will be assessed for quality of reporting using the CHEERS 2022 checklist. Modelling methods will be quality assessed using the appropriate ISPOR principles of good practice. Systematic reviews will be assessed with the PRISMA 2020 Checklist.

PLANNED DATA SYNTHESIS

Strategy for data synthesis
Heterogeneity is expected in the available literature limiting data synthesis. The included studies will be grouped according to each objective corresponding to the three populations;

people diagnosed with GNMLD, carers of people with GNMLD, and people at risk of developing GNMLD. These will be further grouped by intervention and comparator, and type of study (full economic evaluations, partial evaluations, methodology/reviews). All identified economic outcomes will be tabulated, together with the major study characteristics, e.g., population, eligibility criteria, study design, data sources, and interventions. We will report the type of evaluation and methodological approaches. The modelling methodologies will be described, with reported strengths and limitations. The study setting will be reported, with a brief description of the healthcare and social care system, to understand generalisability to other healthcare settings. Patient reported outcomes, health-related quality of life outcomes and utility values, will be tabulated.
If a resource use measure was used for an evaluation, we will report the source of data (patient, patient proxy, medical records, other databases), who completed the measure (patient, their proxy, researcher, health care professional), how it was administered (face-to-face, telephone), and how it was recorded (form, questionnaire, log or diary).
Any inconsistencies, bias or poor quality of studies will be reported. We will assess the relevance to the research questions and the usefulness for decision makers.

Analysis of subgroups or subsets
Evaluations for people diagnosed with GNMLD, carers of people with GNMLD, and people at risk of GNMLD will be assessed separately.

REVIEW AFFILIATION, FUNDING AND PEER REVIEW

Review team members

- Ms Katherine Cullen, Swansea University
- Professor Deborah Fitzsimmons, Swansea University

Review affiliation
Swansea University

Funding source
National Institute for Health and Care Research

Named contact
Katherine Cullen. Swansea Centre for Health Economics, College of Human and Health Sciences, Swansea University, Singleton Park, Swansea, Wales SA2 8PP
katherine.cullen@swansea.ac.uk

TIMELINE OF THE REVIEW

Review timeline
Start date: 30 September 2022. End date: 30 December 2022

Date of first submission to PROSPERO
21 September 2022

Date of registration in PROSPERO
21 September 2022

CURRENT REVIEW STAGE

Publication of review results
The intention is to publish the review once completed.The review will be published in English

Stage of the review at this submission

Review stage	Started	Completed
Pilot work		
Formal searching/study identification		
Screening search results against inclusion criteria		
Data extraction or receipt of IP		
Risk of bias/quality assessment		
Data synthesis		

Review status
The review is currently planned or ongoing.

ADDITIONAL INFORMATION

Additional information
Collaborators

- **Assistant\/Associate Professor Joshua Stott**, University College London

PROSPERO version history

- Version 1.0 published on 21 Sep 2022

Review conflict of interest
None known

Country
Wales

Medical Subject Headings
Caregivers; Cost-Benefit Analysis; Dementia; Humans; Qualitative Research

Disclaimer
The content of this record displays the information provided by the review team. PROSPERO does not peer review registration records or endorse their content.

PROSPERO accepts and posts the information provided in good faith; responsibility for record content rests with the review team. The owner of this record has affirmed that the information provided is truthful and that they understand that deliberate provision of inaccurate information may be construed as scientific misconduct.

PROSPERO does not accept any liability for the content provided in this record or for its use. Readers use the information provided in this record at their own risk.

Any enquiries about the record should be referred to the named review contact
