# Supplementary material for: Development of a resource use measure to capture costs related to unpaid care for people living with non-memory led dementia: a modified Delphi study
Source: BMJ Open. 2026 Feb 12;16(2):e110399. doi: 10.1136/bmjopen-2025-110399 (PMC12911721; doi:10.1136/bmjopen-2025-110399)
Supplement: Supplementary file 4 [file bmjopen-2025-110399.reviewer_comments.pdf]

PEER REVIEW HISTORY

BMJ Open publishes all reviews undertaken for accepted manuscripts. Reviewers are asked to complete a checklist review form and are provided with free text boxes to elaborate on their assessment. These free text comments are reproduced below.

ARTICLE DETAILS

Title (Provisional)

Development of a resource use measure to capture costs related to unpaid care for people living with non-memory led dementia: a modified Delphi study

Authors

Cullen, Katherine; Brotherhood, Emilie V.; Hayes, Oliver; Mansfield, Valerie; Suarez-Gonzalez, Aida; Zimmermann, Nikki; Stott, Joshua; Fitzsimmons, Deborah

---

VERSION 1 - REVIEW

**Reviewer**	**1**
**Name**	**Page, Amy**
**Affiliation**	**The University of Western Australia, Centre for Optimisation of Medicines, School of Allied Health**
**Date**	**25-Oct-2025**
**COI**	**None**

Thank you for the opportunity to review this interesting paper about reaching consensus on the costs to carers of providing care to non-memory led dementias.

Abstract

The abstract was written in a way that made it unclear to the reader what the priority of the research was.

It appears that the researchers found a tool that could be adapted to determine what was important to carers, and then asked health professionals to refine the tool. Once the tools was developed and refined, the authors undertook a modified Delphi (described as a Delphi) to ask 11 health professionals and 9 carers to validate for face validity. The abstract description makes it unclear that this is a tool developed for consumers to reach consensus on the issues that matter to them rather than to support the assertions of the health professionals on what matters to the consumers. I suspect that this was not the intention of the study but the abstract needs to be re-written to ensure that it is clear that it is about working out what is important to the carer rather than the health professionals paternalistically telling the carers what their main issues are.

Methods:

The methods correctly refer to a modified Delphi (whereas the abstract refers to a Delphi). Please update abstract to match the methods.

Inclusion: It is not clear why health professionals are part of the Delphi panel as this is about what matters to consumers.

Sample size: You are correct that there is not consensus on sample size, but the reference cited is old and most samples are larger than 9.

Reviewer 2

Name Vidal, Edison

Affiliation Universidade Estadual Paulista Julio de Mesquita Filho Faculdade de Medicina Campus de Botucatu, Edison I. O. Vidal

Date 04-Dec-2025

COI None

I read this manuscript with interest and hope that my comments below may be useful for the authors in their efforts to strengthen their manuscript.

Major issues

1. Page 6, lines 163 to 164: "Two searches were carried out, the first for people with dementia and their carers was relevant to this study (the full search protocol is available on request)". It is unclear why two searches were conducted instead of one. The authors inform us that one of them was related to people with dementia and their carers, while the other was not relevant for this study. Wouldn't it be better to completely omit the fact that another search unrelated to this study was performed to avoid possible misunderstandings by readers. Furthermore, the authors should make the full search strategy available as an online supplement for the sake of transparency related to the protocol that they registered in the PROSPERO database."

2. Page 6, lines 170 to 172: "On agreement that un-adapted versions of these would not be suitable for inclusion a list of key resource use items was developed using inputs from the identified published evaluations and discussion with key stakeholders…". What do the authors mean by 'un-adapted versions'. Do they mean that resource use measures that were developed for people with dementia in general, i.e., not specifically for people with non-memory led dementias, were discarded?

3. Page 7, lines 190 and 191: "HCPs were recruited from current networks and contacts known to the Chief Investigator and RD-TALK researchers." This kind of recruitment strategy for healthcare professionals seems problematic because it could have led to a less diverse group of participants. The statement made on lines 193 and 194 that 20-30 participants

should be sufficient depends on how participants are selected. Furthermore, that statement was based on the qualitative Delphi method proposed by Dia Sekayi, which was not the methodological approach used in this study. Hence, it seems inappropriate to state that a number of participants greater than 20-30 becomes unmanageable in iterative Delphi studies because it is not uncommon for such studies to include much larger numbers of panelists.

4. Page 7, lines 208 to 211: “Consensus was determined as an IQR of ≤2 units on the Likert scale [26]. The criteria for including an item were ≥70% participants

scoring 7-9 and ≤15% participants scoring 1-3. The criteria for removing items were ≥70% participants scoring 1-3 and ≤15% participants scoring 7-9 [27].” This description seems confusing. There seem to exist two definitions of consensus, one based on the interquartile range and another related to the proportion of participants scoring a certain range of values from Likert scales. Let’s imagine that the median (IQR) for a given item is 5 (4 to 6), which would mean a ‘consensus’ of neutral feelings towards that item, however the same item would not fulfil the criteria do be included or excluded from the final list of consensual items. Moreover, it is very confusing to notice that most items that will not meet the criteria do be included in round 2 will not have met the criteria do be removed, which leads to a paradox for the selection of items to be kept from round 1 to round 2, as one criterium overrides the other. Indeed, this led to further confusion in the interpretation of results.

5. If the statistical analysis plan was established prior to actual analysis, it should be shared as a supplement instead of upon request from authors.

6. Page 8, lines 222 to 227: “We considered the level of agreement across participants and across rounds and documented the degree of stability in scores. The intra-class correlation coefficient (ICC) test (two-way Random effects model with ICC average measurement and absolute agreement between raters) was used to assess inter-rater reliability between rounds for all items to assess agreement among raters with multiple scores for a rater [30]. Agreement of 0.75 or above was taken as an indication of good reliability, 0.5-0.75 as moderate and 0.5 as poor [30]” I am not convinced that it makes sense to assess inter- or intra-rater reliability in a Delphi study because the assumption that responses should be stable does not seem to be appropriate since participants are expected to be able to adjust their opinions over the rounds of the study.

7. Why didn’t the authors include people with Lewy body dementia under the non-memory led dementias? This seems a major limitation of the study.

8. The authors described several different steps/stages for this study. The first stage involved a literature review that was registered in the PROSPERO database as a systematic review. The second stage concerned the development of a list of key resource use items using inputs from the literature identified during the previous stage. The last stage was the Delphi study. The presentation of the results of the review performed in stage I was very brief. It seems that, given the time and resource constraints, the authors had to abandon their intent of

performing a systematic review, which is understandable. However, this should be explained clearly. The reference to the PROSPERO protocol without such an explanation may confuse readers. Nevertheless, since the rapid review was a formal stage of this study, I recommend that the authors provide the details of that review as an online supplement with, for example, the full search strategy and the flowchart of selection of references.

9. The presentation of the results of stage II of the study seems to be restricted to a single paragraph and table. “It was decided to develop a new RUM for this trial, initial items were discussed with the RD-TALK research team and elicited from economic evaluations in dementia as part of the wider review. In an investigation of a service to support carers of people with dementia, the ten tasks from the 2009 Survey of Carers in Households were suggested for the carer tasks [42, 43] (Table 1)”. Reading this, I felt confused because it seemed that, on one hand, the authors stated that they decided to create a new resource use measure but, on the other hand, appear to have used ten tasks present in another instrument that hadn’t been identified in their literature search. The final resou

10. Table 2 is also very confusing. It shows 30 different items and provides information about scores from rounds 1 and 2, including Wilcoxon signed rank sum tests comparing participants’ responses between the two rounds. However, according to the text, only 9 of the original 32 items evaluated in round 1 should be kept in round 2, although no items met the removal criterion, which, very confusingly means that items that should not have remained in round II were kept because they did not meet the removal criterion. Anyway, it looks like 2 items from round 1 were not shown on that table. In addition, the table does not show the 11 new items that were added to round 2 but absent in round 1.

11. Page 14, lines 319 to 321: “Response rates were good for healthcare resources with limited missing data, personal costs were not as well reported, which may be due to non-response if these items were not relevant to the participant.” Does this mean that, when the new instrument was used in the clinical trial, there wasn’t an option for carers do signal which items were not relevant and, therefore, these items appear as missing items?

12. After reading the list of items of the instrument that was presented on table 4, I have the impression that none of the items is specific for people with non-memory led dementias. They seem to apply to people with dementia in general. Isn’t this evidence against the original assumption that specific resource use measures were needed for people with NMLD?

13. It is important that the authors show the complete final version of the instrument that they developed so that others may consider using it in other studies. I am not certain that table 4 fulfills that aim.

14. What does the item “Keeping an eye on him/her to see he/she is all right” mean? Does it mean that the cost of paying for someone to keep an eye on the patient? Or does it mean the time that the carer spent keeping an eye on the patient?

15. Page 13, lines 294-297: “Although speech and language therapy and lasting power of attorney were considered very important in round 2, on discussion with the RD-TALK research team it was decided these items were likely to be for the person with rare dementia and not for the carer and so were excluded from the final RUM.” I understood the reasoning behind the authors’ decision, however I feel unsure that several other items that were mentioned by participants might also have reflected needs and therapies of the people with dementia. For example, on table 3, we can find occupational therapy, neurophysiotherapy, podiatry, aromatherapy and many others that could have been originally directed with regards to patients instead of carers. Moreover, since the stated objectives of the study included identifying candidate resource use measures not only for carers but also for the National Health Service, shouldn’t items directed to patients also be included?

16. Much of the data presented in the first paragraph of the discussion section was not presented in the results section.

17. In the discussion section, the authors mention the difficulty in distinguishing when certain costs/therapies were directed to carers of patients. However, it is unclear how the actual items in the pilot version of the instrument were presented to the carers. It is important to check whether the language that was used was sufficiently clear in distinguishing these activities/costs. Therefore, I recommend sharing both the Delphi questionnaires and the pilot version of the instrument as supplementary materials.

18. The discussion lacks a paragraph dedicated to the limitations of the study. Some limitations are described in a scattered manner in different paragraphs and it is often difficult to be clear if they are being acknowledged as limitations.

Minor issues

a. The “Study Design” subheading should be placed in the methods section of the manuscript.

b. Page 6, lines 155 to 157: “A rapid literature review was conducted to identify evaluations of psychological interventions for people living with dementia and their carers as part of the overall RD-TALK programme grant to determine the most appropriate method for evaluation, as part of the review any RUMs were identified in the literature”. Can the authors revise this sentence to make it clearer.

c. Page 6, line 161. NMLD was mistyped as NLMD.

VERSION 1 - AUTHOR RESPONSE

Reviewer: 1
Dr. Amy Page, The University of Western Australia
Comments to the Author:
Thank you for the opportunity to review this interesting paper about reaching consensus on the costs to carers of providing care to non-memory led dementias.

Abstract

The abstract was written in a way that made it unclear to the reader what the priority of the research was.
It appears that the researchers found a tool that could be adapted to determine what was important to carers, and then asked health professionals to refine the tool. Once the tools was developed and refined, the authors undertook a modified Delphi (described as a Delphi) to ask 11 health professionals and 9 carers to validate for face validity. The abstract description makes it unclear that this is a tool developed for consumers to reach consensus on the issues that matter to them rather than to support the assertions of the health professionals on what matters to the consumers. I suspect that this was not the intention of the study but the abstract needs to be re-written to ensure that it is clear that it is about working out what is important to the carer rather than the health professionals paternalistically telling the carers what their main issues are.

- Thank you for you comments. The abstract has been re-written to be clearer and to highlight the involvement of carers.

Methods:
The methods correctly refer to a modified Delphi (whereas the abstract refers to a Delphi). Please update abstract to match the methods.

- Thank you for your comment the abstract has been updated.

Inclusion: It is not clear why health professionals are part of the Delphi panel as this is about what matters to consumers.

We will be collecting resource use data in the BELIDE trial to inform costs in the economic evaluation. From the discussions with the research team including the patient and public involvement representatives, the primary perspective will be societal including personal costs as well as health and social care. The economic evaluation will be designed to be used by various stakeholders, public and patients, as well as healthcare professionals. Therefore it is important that the measure development is informed by healthcare professionals as well as carers. I've added about cognitive diversity in the panel into the methods section (p6 lines 181-183 clean version).

Sample size: You are correct that there is not consensus on sample size, but the reference cited is old and most samples are larger than 9.

When we have carried out modified delphi studies for resource use measures in other conditions we have always attempted to get more patient participants than healthcare professionals as they are able to present a more complete picture of their resource use and costs. With this study we struggled to get carer engagement, it may have been the timing of the study, and also this group of carers are younger, likely working and with other family commitments. I have added the small numbers of carers as a limitation in the discussion and added more explanation of the sample size in the methods section, with more recent references (p7 lines 204-213, p17 lines 384-385).

Reviewer: 2
Prof. Edison Vidal, Universidade Estadual Paulista Julio de Mesquita Filho Faculdade de Medicina Campus de Botucatu
Comments to the Author:
I read this manuscript with interest and hope that my comments below may be useful for the authors in their efforts to strengthen their manuscript.

Major issues

1. Page 6, lines 163 to 164: “Two searches were carried out, the first for people with dementia and their carers was relevant to this study (the full search protocol is available on request)”. It is unclear why two searches were conducted instead of one. The authors inform us that one of them was related to people with dementia and their carers, while the other was not relevant for this study. Wouldn’t it be better to

completely omit the fact that another search unrelated to this study was performed to avoid possible misunderstandings by readers. Furthermore, the authors should make the full search strategy available as an online supplement for the sake of transparency related to the protocol that they registered in the PROSPERO database."

Thank you for your comment. I have removed reference to the second search and will include the PROSPERO protocol as supplementary material.

2. Page 6, lines 170 to 172: "On agreement that un-adapted versions of these would not be suitable for inclusion a list of key resource use items was developed using inputs from the identified published evaluations and discussion with key stakeholders…". What do the authors mean by 'un-adapted versions'. Do they mean that resource use measures that were developed for people with dementia in general, i.e., not specifically for people with non-memory led dementias, were discarded?

Thank you for your comment, there was a measure identified for dementia which was reviewed by the PPI member of the research team. They felt that some items were not relevant and there were items missing and so it was decided to do this further work. I have changed this to be clearer - 'on agreement that there were no suitable resource use measures available for this population...'

3. Page 7, lines 190 and 191: "HCPs were recruited from current networks and contacts known to the Chief Investigator and RD-TALK researchers." This kind of recruitment strategy for healthcare professionals seems problematic because it could have led to a less diverse group of participants. The statement made on lines 193 and 194 that 20-30 participants should be sufficient depends on how participants are selected. Furthermore, that statement was based on the qualitative Delphi method proposed by Dia Sekayi, which was not the methodological approach used in this study. Hence, it seems inappropriate to state that a number of participants greater than 20-30 becomes unmanageable in iterative Delphi studies because it is not uncommon for such studies to include much larger numbers of panelists.

I've added in some detail of the Rare Dementia Support as it is the primary service for people living with rare dementia in the UK (also with international members) and details of recruitment of HCPs from the protocol for this study. For rare conditions it is more difficult to recruit HCPs with enough experience to understand the needs of carers for people living with a rare dementia. I have included some explanation as to why the recruitment centred around the RDS. (p7 lines 186 - 202 clean version)

I have removed the sentence on the unmanageability of larger panels in a Delphi, and have referred to the recruitment challenges in rare diseases which was our main reason for limiting the numbers included. This resource use measure was developed alongside two other measures, and other parts of the research programme which made recruitment more difficult. (p7 lines 194-202 clean version).

4. Page 7, lines 208 to 211: "Consensus was determined as an IQR of ≤2 units on the Likert scale [26]. The criteria for including an item were ≥70% participants scoring 7-9 and ≤15% participants scoring 1-3. The criteria for removing items were ≥70% participants scoring 1-3 and ≤15% participants scoring 7-9 [27]." This description seems confusing. There seem to exist two definitions of consensus, one based on the interquartile range and another related to the proportion of participants scoring a certain range of values from Likert scales. Let's imagine that the median (IQR) for a given item is 5 (4 to 6), which would mean a 'consensus' of neutral feelings towards that item, however the same item would not fulfil the criteria do be included or excluded from the final list of consensual items. Moreover, it is very confusing to notice that most items that will not meet the criteria do be included in round 2 will not have met the criteria do be removed, which leads to a paradox for the selection of items to be kept from round 1 to round 2, as one criterium overrides the other. Indeed, this led to further confusion in the interpretation of results.

The resource use measure is often the longest questionnaire included and to make sure we are including the most important resource items, and that the questionnaire is not too long as to be a burden to the research participants, we set criteria for inclusion of items and exclusion. Consensus could be reached on an item without it reaching the criteria for inclusion or exclusion, but this does demonstrate stability in the responses. I have re-written this section to be clearer on why two measures were used. I have also moved the consensus explanation as all items were included in both rounds to allow respondents to see the overall rankings and make changes, or not, to their own ranking. (p8 lines 222-230 clean version)

5. If the statistical analysis plan was established prior to actual analysis, it should be shared as a

supplement instead of upon request from authors.

The analysis plan will be made available as supplementary material.

6. Page 8, lines 222 to 227: "We considered the level of agreement across participants and across rounds and documented the degree of stability in scores. The intra-class correlation coefficient (ICC) test (two-way Random effects model with ICC average measurement and absolute agreement between raters) was used to assess inter-rater reliability between rounds for all items to assess agreement among raters with multiple scores for a rater [30]. Agreement of 0.75 or above was taken as an indication of good reliability, 0.5-0.75 as moderate and 0.5 as poor [30]" I am not convinced that it makes sense to assess inter- or intra-rater reliability in a Delphi study because the assumption that responses should be stable does not seem to be appropriate since participants are expected to be able to adjust their opinions over the rounds of the study.

We wanted to assess the consistency of the responses from one round to another, if there was a lot of change from one round to the next. Although participants are able to adjust their opinions over the rounds of the study we are looking to get to the point where their opinions stop changing to make the decision on what is included in the final resource use measure.

7. Why didn't the authors include people with Lewy body dementia under the non-memory led dementias? This seems a major limitation of the study.

We designed this measure with people with dementia types where specific cognitive functions other than memory are the initial functions affected, this was in part because this measure was designed in the context of trials for these specific populations. However, we recognise that memory, is typically less affected than other abilities early in the course of Lewy Body dementia and have put this as a limitation. This has been included as a limitation in the discussion section.(p17 lines 285-290 clean version)

8. The authors described several different steps/stages for this study. The first stage involved a literature review that was registered in the PROSPERO database as a systematic review. The second stage concerned the development of a list of key resource use items using inputs from the literature identified during the previous stage. The last stage was the Delphi study. The presentation of the results of the review performed in stage I was very brief. It seems that, given the time and resource constraints, the authors had to abandon their intent of performing a systematic review, which is understandable. However, this should be explained clearly. The reference to the PROSPERO protocol without such an explanation may confuse readers. Nevertheless, since the rapid review was a formal stage of this study, I recommend that the authors provide the details of that review as an online supplement with, for example, the full search strategy and the flowchart of selection of references.

Thank you for your comment. The PRISMA diagram will be included as supplementary material and the PROSPERO protocol which includes the full search strategy.

9. The presentation of the results of stage II of the study seems to be restricted to a single paragraph and table. "It was decided to develop a new RUM for this trial, initial items were discussed with the RD-TALK research team and elicited from economic evaluations in dementia as part of the wider review. In an investigation of a service to support carers of people with dementia, the ten tasks from the 2009 Survey of Carers in Households were suggested for the carer tasks [42, 43] (Table 1)". Reading this, I felt confused because it seemed that, on one hand, the authors stated that they decided to create a new resource use measure but, on the other hand, appear to have used ten tasks present in another instrument that hadn't been identified in their literature search. The final resou

I have added some more explanation here (p9, lines 270-277). In a published assessment of a service to support carers of people with dementia they used the ten tasks from the Survey of Carers in Households for reporting of carer tasks as well as asking about health and social care resource use. The full list of questions included was not published and only overall healthcare costs were presented. As an initial list for the modified Delphi study we used these tasks, the research team adding three additional carer tasks. It will be important for the economic evaluations to take into account unpaid caring time. The RUD has three carer task categories:
How much time per day did you spend assisting the patient with tasks such as toilet visits, eating,

dressing, grooming, walking and bathing?
How much time per day did you spend assisting the patient with tasks such as shopping, food preparation, housekeeping, laundry, transportation, taking medication and managing financial matters?
How much time per day did you spend supervising the patient (i.e. preventing dangerous events)?
When we asked the RD-talk PPI to review the RUD questionnaire the carers tasks were questioned, likely due to the questionnaire being tailored to more common forms of dementia with different symptoms. During the modified Delphi process the carers only rated three of the ten tasks as very important - help dealing with care service and benefits, other paperwork or financial matters, and giving medicines. Asking unpaid carers to specify tasks related to caring can be subjective, having a initial list helped us to be consistent in what tasks were considered. It is helpful to have an initial list that respondents can comment on and add to, and this standard list was identified as a good starting point.

10. Table 2 is also very confusing. It shows 30 different items and provides information about scores from rounds 1 and 2, including Wilcoxon signed rank sum tests comparing participants' responses between the two rounds. However, according to the text, only 9 of the original 32 items evaluated in round 1 should be kept in round 2, although no items met the removal criterion, which, very confusingly means that items that should not have remained in round II were kept because they did not meet the removal criterion. Anyway, it looks like 2 items from round 1 were not shown on that table. In addition, the table does not show the 11 new items that were added to round 2 but absent in round 1.

In this modified Delphi all items were presented in round 1, and then in round all items were presented again with the rankings from round 1 to allow people to change their rankings. I have changed the explanation of the methodology to make it clearer that the criteria for inclusion and exclusion relate to the final measure. The percentage agreement has been presented for both rounds to allow readers to see how the rankings changed.

11. Page 14, lines 319 to 321: "Response rates were good for healthcare resources with limited missing data, personal costs were not as well reported, which may be due to non-response if these items were not relevant to the participant." Does this mean that, when the new instrument was used in the clinical trial, there wasn't an option for carers do signal which items were not relevant and, therefore, these items appear as missing items?

Each item in the measure has a yes or no option. For the productivity questions related to working, there is a branching question asking whether someone is retired, self-employed or not working. The final resource use measure will be made available as supplementary material.

12. After reading the list of items of the instrument that was presented on table 4, I have the impression that none of the items is specific for people with non-memory led dementias. They seem to apply to people with dementia in general. Isn't this evidence against the original assumption that specific resource use measures were needed for people with NMLD?

This measure may also be useful for people living with more common types of dementia. But the research team, including a carer representative, felt that the measures designed for people living with dementia, not specifically rare dementias, were not suitable for this population.

13. It is important that the authors show the complete final version of the instrument that they developed so that others may consider using it in other studies. I am not certain that table 4 fulfills that aim.

The final resource use measure will be included in the supplementary material

14. What does the item "Keeping an eye on him/her to see he/she is all right" mean? Does it mean that the cost of paying for someone to keep an eye on the patient? Or does it mean the time that the carer spent keeping an eye on the patient?

In the final measure we are asking about time the carer spent on these tasks, not employment of a paid carer to do the tasks. This can then be used to develop costs related to informal care. The resource use measure will be used in a trial for an intervention for unpaid carers so we are interested in the unpaid care only.

15. Page 13, lines 294-297: "Although speech and language therapy and lasting power of attorney were considered very important in round 2, on discussion with the RD-TALK research team it was decided these items were likely to be for the person with rare dementia and not for the carer and so were excluded from the final RUM." I understood the reasoning behind the authors' decision, however I feel unsure that several other items that were mentioned by participants might also have reflected needs and therapies of the people with dementia. For example, on table 3, we can find occupational therapy, neurophysiotherapy, podiatry, aromatherapy and many others that could have been originally directed with regards to patients instead of carers. Moreover, since the stated objectives of the study included identifying candidate resource use measures not only for carers but also for the National Health Service, shouldn't items directed to patients also be included?

Of the new items suggested in round 1, only these two were considered very important in round 2 whereas the other items were not rated as highly (p13 lines 308-311). The intervention being assessed in the BELIDE study is for carers of people living with a rare dementia and so the focus is on the carers outcomes. It is a psychological intervention and unlikely to impact the resource use requirements for the person living with dementia. The NHS costs are being collected mainly for primary care as that was considered most relevant for carers. Another trial of an intervention for people living with a rare dementia is also being conducted where the resources items related to the person living with dementia will be collected. It is important that the RUM is not too long as to be a burden to the carers participating in the trial, and to ask about all healthcare use related to the person with dementia as well as the carer would result in a very long measure.

16. Much of the data presented in the first paragraph of the discussion section was not presented in the results section.

These results are presented in lines 316 to 328 on page 14.

17. In the discussion section, the authors mention the difficulty in distinguishing when certain costs/therapies were directed to carers of patients. However, it is unclear how the actual items in the pilot version of the instrument were presented to the carers. It is important to check whether the language that was used was sufficiently clear in distinguishing these activities/costs. Therefore, I recommend sharing both the Delphi questionnaires and the pilot version of the instrument as supplementary materials.

The final measure will be included as supplementary material and the first version of the delphi questionnaire.

18. The discussion lacks a paragraph dedicated to the limitations of the study. Some limitations are described in a scattered manner in different paragraphs and it is often difficult to be clear if they are being acknowledged as limitations.

The limitations have been made clearer the discussion, with the main limitations of the process brought in one paragraph (p17 lines 384-390).

Minor issues

The following minor issues have been corrected.

a. The "Study Design" subheading should be placed in the methods section of the manuscript.

b. Page 6, lines 155 to 157: "A rapid literature review was conducted to identify evaluations of psychological interventions for people living with dementia and their carers as part of the overall RD-TALK programme grant to determine the most appropriate method for evaluation, as part of the review any RUMs were identified in the literature". Can the authors revise this sentence to make it clearer.

c. Page 6, line 161. NMLD was mistyped as NLMD.
